# Posterior-Chamber Phakic Intraocular Lens Implantation in Patients over 40 Years of Age

**DOI:** 10.1155/2020/7457902

**Published:** 2020-06-29

**Authors:** Pedro Tañá-Rivero, Francisco Pastor-Pascual, Marceliano Crespo, José L. Rodríguez-Prats, José J. Muñoz-Tomás, Robert Montés-Micó

**Affiliations:** ^1^Oftalvist Clinic, Spain; ^2^Optics and Optometry & Vision Sciences Dept, University of Valencia, Valencia, Spain

## Abstract

**Purpose:**

To assess the efficacy, safety, and predictability of the Visian Implantable Collamer Lens (ICL) model having a central port in patients over 40 years of age.

**Methods:**

This study included 33 eyes from 21 patients who underwent V4c ICL implantation for the correction of myopia and myopic astigmatism. We assessed uncorrected (UDVA) and corrected (CDVA) distance visual acuity, refraction, intraocular pressure (IOP), endothelial cell density (ECD), vault, and adverse events occurring over a 1-year period.

**Results:**

Mean age of the patients at the time of implantation was 43.52 ± 4.49 years (range: 40 to 56 years). Efficacy and safety indexes were 1 and 1.09, respectively. Surgical outcomes for CDVA were as follows: no eye lost any lines, 19 eyes (57.58%) showed no CDVA changes, 7 eyes (21.21%) gained 1 line, 4 eyes (12.12%) gained 2 lines, and 3 eyes (9.09%) gained ≥3 lines. Mean postoperative spherical equivalent (SE) was −0.09 ± 0.47 D. A total of 29 eyes (87.8%) were within ±0.50 D and 31 eyes (93.9%) were within ±1.00 D of the desired SE. At 1-year, mean IOP was 15.27 ± 3.03 mmHg (range: 9 to 20 mmHg, *p*=0.12 pre vs. post) and mean ECD was 2516 ± 234 cells/mm^2^ (*p*=0.29 pre vs. post). Mean postoperative vault was 320 ± 136 *μ*m, with 201–300 *μ*m being the most prevalent vault range for 9 eyes (31.03%). None of the eyes showed a vault >701 *μ*m. There were neither intraoperative nor postoperative complications; in fact, all ICL implantation procedures were uneventful.

**Conclusions:**

Our study's findings support the use of this lens in patients over 40 years of age. A long follow-up period is advisable to monitor ICL position relative to the crystalline lens.

## 1. Introduction

The use of phakic intraocular lenses is an approach that is frequently used to correct different amounts of refractive errors. However, it is highly myopic patients—with or without astigmatism—who have especially benefited from the good visual and refractive outcomes that these intraocular lenses provide. One of the most popular phakic lenses is the posterior-chamber phakic Visian Implantable Collamer Lens (ICL) (Staar Surgical AG, Nidau, Switzerland), whose latest model (Visian V4c) incorporates a 0.36 mm central port (i.e., a hole) (KS-Aquaport). This opening allows the physiological flow of aqueous, thus making Nd : YAG peripheral iridotomies or intraoperative iridectomies unnecessary. Recent reviews and meta-analysis have confirmed its safety and effectiveness in moderately and highly myopic eyes throughout short, medium, and long follow-up periods [1, 2]. With these outcomes, and bearing in mind that no vision-threatening complications occurred, the use of this IOL is recommended for the correction of a wide range of myopic errors.

However, few studies have included in their samples patients older than 40 years of age, and when they did so, the data analysis encompassed the whole sample, thus including patients of different ages (usually cohorts between 20 and 45 years). Consequently, specific studies are needed where this lens' performance is assessed as a function of the patient's age. Aging and, more specifically, the age-related changes that happen in the crystalline lens (i.e., increase in thickness) is an important factor to be considered when ICLs are implanted in people belonging to this age group. Two recent publications by the same group [3, 4] showed the outcomes for early presbyopic patients (aged 40 to 53; 6 months of follow-up period) but with a slight intentional postoperative undercorrection [3, 4]. They concluded that is a viable surgical presbyopic treatment for this type of patients. Nonetheless, we believe that the use of these lenses in patients over 40 requires a more specific analysis so as to fully understand their clinical performance. Hence, the objective of this study was to assess the visual and refractive outcomes of the V4c Visian ICL model for the correction of myopia and astigmatism when implanted in patients over 40 years of age.

## 2. Material and Methods

### 2.1. Patients

A total of 33 eyes from 21 patients (6 males and 15 females) aged 40 to 56 were consecutively recruited for this prospective study. These patients underwent the implantation of the Visian V4c ICL to have their myopia and their myopic astigmatism corrected at the Oftalvist Clinic. The procedures took place between March 2017 and February 2019. The inclusion criteria were age ≥40 years, and myopic and astigmatic refractive error to be corrected with the V4c ICL. The exclusion criteria were endothelial cell density (ECD) below 2000 cell/mm^2^, anterior chamber depth (ACD) < 2.8 mm, cataract, and a history of glaucoma or retinal detachment, neuro-ophthalmic diseases, macular degeneration, retinopathy, or any inflammatory disease. The study adhered to the tenets of the Declaration of Helsinki and was approved by the Institutional Review Board.

### 2.2. Preoperative Examination

The preoperative examination included uncorrected distance visual acuity (UDVA), corrected distance visual acuity (CDVA), manifest refraction, slit-lamp, and a fundus examination carried out with ophthalmoscopy and using the Cirrus HD-OCT 5000 (Carl Zeiss Meditec AG, Jena, Germany). Moreover, Goldmann applanation tonometry was used to measure intraocular pressure (IOP) and ECD was measured with specular microscopy (SP-1P, Topcon, Tokyo, Japan). Lastly, for the anterior-segment analysis, the ANTERION Swept-Source Optical Coherence Tomography platform (SS-OCT, Heidelberg Engineering, Inc, Heidelberg, Germany) was used to measure ACD, white-to-white (WTW) distance, and angle-to-angle (ATA) distance and for keratometry.

### 2.3. Phakic Intraocular Lens Selection

All eyes included in this study had either myopic or toric V4c ICLs implanted. ICL power calculation was carried out using a modified vertex formula according to the manufacturer's instructions. All eyes were targeted for emmetropia. ICL size calculation was based on the horizontal WTW, ACD, and ATA distances that had been measured with the ANTERION SS-OCT. Topical antibiotic agents were used for 2 days before surgery. On the day of surgery, patients were administered tropicamide eyedrops. Following topical anaesthesia, the ICL was introduced into the anterior chamber through a 2.75 mm temporal corneal incision or on the steepest meridian for astigmatism <1.00D using a STAAR injector and cartridge (Staar Surgical AG, Nidau, Switzerland) once the chamber had been filled with a viscosurgical device (Provisc, Alcon Laboratories Inc, Fort Worth, TX, USA). This viscosurgical device was completely removed at the end of the procedure. Once surgery was completed, dexamethasone and antibiotic medications were topically administered 5 times per day for 3 days, 3 times per day for 2 weeks, with the dose being reduced gradually thereafter. Follow-up visits were scheduled at 1 day, 5 days, and 1, 4, and 12 months postoperatively.

### 2.4. Postoperative Examination

Postoperative data were collected at the follow-up visits occurring at 1 day, 5 days, and 1, 4, and 12 months. For the purpose of this study, UDVA, CDVA, manifest refraction, IOP, and ECD values obtained at the 1-year follow-up visit were compared with preoperative values. Moreover, central vault height (i.e., the distance from the ICL's posterior surface to the crystalline lens' anterior surface) was also measured with the ANTERION SS-OCT as part of the postoperative examination. The same examiner performed all examinations.

### 2.5. Outcomes Analysis

Preoperative and 1-year postoperative visual acuity values—both UDVA and CDVA—were used to assess the efficacy and the safety of the procedure. As for predictability, the endpoint was the attempted-versus-achieved manifest refraction. For this purpose, manifest refraction in conventional script notation (S [sphere], C [cylinder], a [axis]) was converted into power vector coordinates: SE = *S* + C/2, *J*_0_ = (−C/2) *X* cos (2*α*), and *J*_45_ = (−C/2) *X* sin (2*α*), where SE is the spherical equivalent and *J*_0_ and *J*_45_ are the two Jackson crossed-cylinders equivalent to the conventional cylinder. The efficacy index (defined as the ratio between postoperative UDVA and preoperative CDVA) and the safety index (defined as the ratio between postoperative CDVA and preoperative CDVA) were calculated based on Snellen decimal visual acuity values. Data analysis was performed using Excel (2016 version, Microsoft Corporation, Redmond, WA, USA). The Wilcoxon signed-rank test was used to perform the preoperative-vs-postoperative data comparison. The results shown represent mean ± standard deviation (SD) values; a *p* value below 0.05 was considered to be statistically significant.

## 3. Results

The study comprised 33 eyes from 21 patients. Mean age of the 6 men (28.6%) and 14 women (71.4%) was 43.52 ± 4.49 years (range from 40 to 56 years). Mean preoperative SE refractive error was −8.46 ± 3.31 D (range: −14.88 to −1.88 D).  Table 1 shows a summary of preoperative patient demographic data and implanted ICL characteristics. A toric ICL was implanted in 10 eyes (30.3%); those were the ones requiring astigmatism correction of 1.5 D or more. Regarding ICL diameter, 2 eyes were implanted with the 12.1 model, 9 eyes with the 12.6 model, and 22 eyes with the 13.2 model. Visual acuity outcomes, refractive error with SE and astigmatism vector analysis, IOP, ECD, vault, and adverse events were evaluated. The standard graphs for reporting refractive and visual acuity outcomes were constructed for each parameter analysed.

### 3.1. Visual Acuity Outcomes: Safety and Efficacy

**Figure 1:**
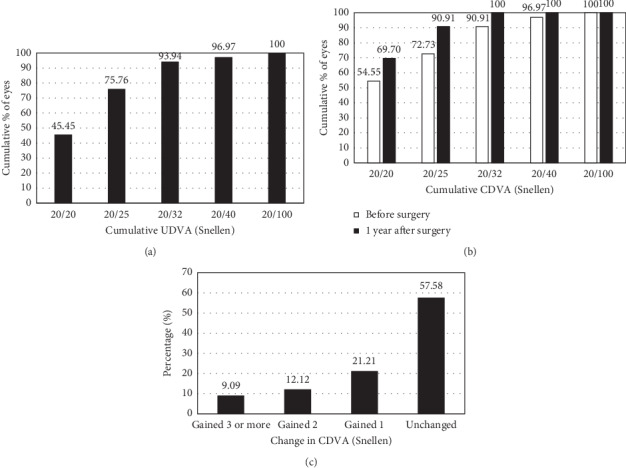
Cumulative uncorrected distance visual acuity (UDVA), at 1 year after surgery (a), cumulative corrected distance visual acuity (CDVA) before and at 1 year after surgery (b), and postoperative changes in corrected distance visual acuity (CDVA) at 1 year after surgery (c).

At 1 year, mean postoperative Snellen decimal UDVA and CDVA were 0.88 ± 0.16 and 0.96 ± 0.09, respectively.  Figure 1 shows the cumulative UDVA at 1 year after surgery (A, top), and CDVA before and 1 year after surgery (B, middle). As can be seen in those graphs, at 1 year a total of 25 eyes (75.76%) and 30 eyes (90.91%) had achieved an UDVA and CDVA of 20/25 or better, respectively.  Figure 1 (C, bottom) illustrates the postoperative changes in CDVA at 1 year (relative to preoperative values). None of the eyes lost any lines, 19 eyes (57.58%) maintained their preoperative CDVA, 7 eyes (21.21%) gained 1 line, 4 eyes (12.12%) gained 2 lines, and 3 eyes (9.09%) gained 3 or more lines of CDVA. The resulting efficacy and safety indexes were 1 and 1.09, respectively.

### 3.2. Predictability

**Figure 2:**
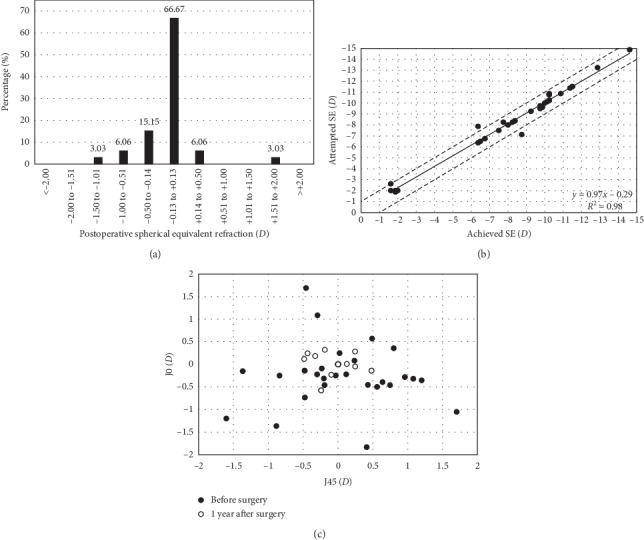
Postoperative spherical equivalent accuracy (a), attempted-versus-achieved spherical equivalent (b), and scatterplot of preoperative and 1-year postoperative astigmatic vectors (c). Solid line in figure B represents the best-fit line (regression equation and R value was included) and dotted lines ±1.00D. In the third figure, the more central location of postoperative data around 0 represents a reduction in astigmatism after lens implantation (*J*0 = Jackson cross-cylinder, axes at 180° and 90°; J45 = Jackson cross-cylinder, axes at 45° and 135°).

Figure 2(a) illustrates postoperative SE accuracy measured 1 year after surgery; in particular, it shows that for 22 eyes (66.7%) SE refraction was within the ±0.13 D range (emmetropia). Mean postoperative SE was −0.09 ± 0.47 D.  Figure 2(b) is a scatterplot of the attempted-versus-achieved SE at 1 year after surgery. 29 eyes (87.8%) were within ±0.50 D and 31 eyes (93.9%) were within ±1.00 D.  Figure 2(c) shows the astigmatic component of the power vector as represented by a 2-dimensional vector (J0, J45). In this graph (0,0) the origin represents an astigmatism-free eye. The spread observed in the presurgical data becomes a concentrated data set around the origin after lens implantation. Note that the central open circle located at (0, 0) represents 23 eyes that ended up having 0 D of postoperative astigmatism.

### 3.3. Intraocular Pressure, Endothelial Cell Density, and Vault

**Figure 3:**
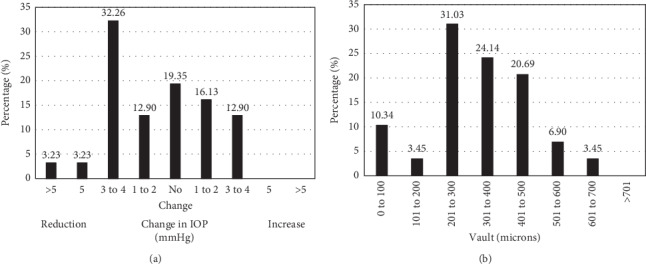
Postoperative changes in intraocular pressure (IOP), measured in mmHg, at 1 year after surgery (a), and distribution of eyes according to the vault, measured in microns, at 1 year after surgery (b).

Mean IOP at 1 year after surgery was 15.27 ± 3.03 mmHg (range: 9 to 20 mmHg, *p*=0.12 pre vs. post).  Figure 3(a) shows the postoperative change in IOP after ICL implantation. It is worth pointing out that no increase exceeding 4 mmHg occurred in any case during the follow-up and that more than 50% of the eyes experienced a reduction in IOP (51.62%). As for the ECD, mean ECD at 1 year was 2516 ± 234 cells/mm^2^; the change relative to preoperative values did not turn out to be statistically significant (Table 1, *p*=0.29 pre vs. post). More specifically, mean change in ECD ((preoperative ECD–postoperative ECD) × 100/preoperative ECD) was 2.04%. Lastly, mean postoperative vault was 320 ± 136 *μ*m at 1 year (range: 96 to 603 *μ*m).  Figure 3(b) shows the postoperative distribution of these vault values. The most prevalent vault range was from 201 to 300 *μ*m (9 eyes; 31.03%) followed by the 301 to 400 *μ*m range (7 eyes; 24.14%). No eyes showed a vault larger than 701 *μ*m.

### 3.4. Adverse Events

There were not any complications (neither intraoperative nor postoperative ones); in fact, all ICL implantations were uneventful. Over 1 year of follow-up, no cases of anterior subcapsular opacity, cataract, pigment dispersion glaucoma, pupillary block, or other vision-threatening complications were reported.

## 4. Discussion

In the present study, we have assessed the visual and refractive 1-year postoperative outcomes yielded by the V4c ICL model in a group of patients aged over 40. The objective of the present study was to find out whether this model is a safe, effective, and predictable option to correct myopia and myopic astigmatism in this cohort of patients.

The visual outcomes found in our series were satisfactory in terms of safety and efficacy indexes. Namely, the safety index was 1.09, and none of the eyes had lost any line of CDVA, whereas 42.42% of them gained 1 line or more of CDVA (Figure 1(c)). Most of the eyes maintained their CDVA (57.58%). The percentage of eyes with 20/20 CDVA increased from 54.55% preoperatively to more than 69.70% postoperatively (Figure 2(b)). Similarly, there was also an increase for the 20/25 category, namely, from 72.73% preoperatively to 90.91% after surgery. As for the efficacy index, it was 1. Note also that most eyes had an UDVA of 20/25 or better (>75%, Figure 1(a)). Like the one we have introduced, there are different studies in the literature analysing this lens with short, medium, and long follow-up periods [1, 2]. In order to compare our outcomes with those published previously, we have considered for the discussion only those studies reporting data for the V4c ICL model at 1 year of follow-up. To do that, a literature review search was carried out including the following databases: PubMed (US National Library of Medicine), Web of Science (Thomson Reuters), and Scopus (Elsevier, BV). The search was limited to publications written in English. The date of the last electronic search was March 24, 2020. The literature search based on these criteria resulted in 8 relevant contributions being considered [5–12].  Table 2 summarizes the information about the sample size (number of eyes), patients' age, and SE values of these studies. In addition to this, Table 3 shows these studies' visual outcomes. For example, our safety and efficacy indexes were similar to those reported by Lisa et al. [5], 1.04 and 1.0, respectively, and slightly different from other authors'. Note that in Lisa et al.'s study [5] their preoperative SE was similar to our sample's (about −8.50 D). However, we want to point out that, in addition to possible differences across studies in terms of preoperative SE values, mean age and age range also varied significantly across studies (see Table 2). For instance, mean age and SE in Sachdev et al.'s [12] study were 24 years and −9.44 D, whereas in Kamiya et al.'s [9], for the low-to-moderate myopia group, they were 34.8 years and −4.29 D, respectively.

If we focus now on predictability, we can consider these procedures to be successful, since 90.6% and 93.9% of the eyes were within the ±0.50 and ± 1.00 D range of postoperative refractive error, respectively. Both values were also comparable and sometimes better (versus Ganesh et al. [7], García-de-la-Rosa et al. [10], and Niu et al. [11]) than those found in previous studies (see Table 3). Similarly, SE was also good, being below a quarter of diopter (−0.09 ± 0.47D). From the same figure, we may observe that 22 eyes (66.7%) were within ±0.13D (*A*) and a high concentration of points around the origin of the graph (0, 0) corresponding to a value of 0D of astigmatism (C). The patients included in our sample were about 10 years older than those in Kamiya et al.'s sample [9] (this was the study with the highest mean age), whereas mean SE and percentage of eyes ±0.50 and ± 1.00 D were comparable. Taking into account our outcomes and those previously published with different samples, our results are similar and showed the excellent predictability of the procedure in these patients. The age of the patient does not seem to be a factor that has an impact upon the procedure's predictability.

Some surgeons may consider implanting this lens following a monovision or undercorrection strategy. This approach was attempted by Kamiya et al. [3] and Takahashi et al. [3]. The first study assessed 34 eyes, mean patients' age was 46.1 years (from 40 to 53 years), and mean SE was −8.67 D (from −2.25 to −18.25 D); the second study included 42 eyes, mean patients' age was 45 years (from 40 to 53 years), and mean SE was −7.37 D (from −2.25 to −14.75 D). Both studies reported their outcomes at 6 months after surgery. The first study selected emmetropia in the dominant eye and −0.5 to −1.0 D in the nondominant eye as their target refraction, whereas the second study approximately −0.50 to −1.50 D in both eyes. They reported that 100% of eyes were within the ±0.50 D range and the mean UDVA and CDVA were about −0.04 and −0.19 logMAR, respectively. Both studies concluded that monovision [3] or intentional undercorrection [4] provided good binocular vision at near to far distances, suggesting its feasibility as a new surgical presbyopic approach for early presbyopia. We have not considered this option in our series.

Regarding IOP, in our study mean IOP was 15.27 ± 3.03 mmHg (range: 9 to 20 mmHg), which is similar to that found before surgery (*p*=0.12).  Figure 3(a) shows the postoperative change in IOP after ICL implantation, and from this figure we may observe that 51.62% of eyes showed a reduction, 19.35% of eyes no change, and 29.03% of eyes a mild increase in IOP compared to preoperative values. No acute postoperative hypertension and no increase of more than 4 mmHg occurred in any case during the follow-up. Our results (mean IOP = 15.27 ± 3.03 mmHg) are in good agreement with those found by other authors at 1 year (about 15 mmHg, see Table 3). The central port of the lens, designed to allow physiologic flow of aqueous without resorting to Nd : YAG peripheral iridotomies or intraoperative iridectomies, confirms that IOP was well controlled after surgery. However, our follow-up of 1 year is short and these patients should be kept under follow-up for longer periods. Shimizu et al. [13] and Alfonso et al. [14] with 5 years of follow-up in younger patients implanted with this lens followed this approach of extended follow-up period (mean ages of 31.9 and 31.2 years, respectively). Our mean ECD at 1 year showed no statistically significant differences with their corresponding preoperative values (2516 ± 234 vs. 2568 ± 332 cells/mm^2^, *p*=0.29). These mean values were similar to those found in previous studies, which ranged from 2512 [8] to 2963 [11] cells/mm^2^. The percentage loss in our sample amounted to 2.04%, whereas in previous studies it was rather diverse, ranging from 1.7% [5] to 9.0% [7]. A physiologic cell loss of approximately 0.6% per year [15, 16] is to be expected, although higher values of loss may happen at early times related to the surgical procedure.

As for vault, we have found a mean vault of 320 *μ*m in our patients.  Figure 3(b) shows the exact distribution of vault per interval, the 201–300 *μ*m interval being the range corresponding to the highest percentage of eyes (31.03%). Few studies in the literature reported vault values (see Table 3); however, those studies that measured it showed values ranging from 380 [11] to 628 *μ*m [6], which are slightly higher than what we found in our study; this could be connected with the changes that happen in the crystalline lens with aging. Vault is negatively correlated with age [17]. ACD decreases with age because of the thickening of the aging crystalline lens [18], which results in a lower vault. Such decrease in ACD is likely to be induced by the thickening of the aging crystalline lens, which happens at an average rate of 24 *μ*m/year [19]. A review of potential complications of ICL implantation concluded that low vault is related to cataract formation [20] and the rate of vault reduction is faster in patients who developed lens opacity [21, 22]. The change in vault with time has been also reported in a long follow-up study with this specific model. The 5-year study carried out by Alfonso et al. [14] showed a reduction of about 1.2 *μ*m/month. Changes with time (due to crystalline lens thickening) should be always borne in mind, especially in patients with low vault values.

No postoperative adverse events occurred during the 1 year of follow-up. Previous studies have described some postoperative complications that include ECD loss, IOP increase, or cataracts [20]. It seems that cataract formation is the most frequent complication after ICL implantation; this may be linked to the patient's age at the time of surgery. Lackner et al. [23] indicated that the most important factor that influences the likelihood of crystalline lens opacification was the age of the patient, and potentially, its vulnerability and susceptibility increase with advanced age. They reported in a series of 76 eyes that all occurrences of late cataract development (*n* = 11) were in patients older than 50 years of age. Similarly, Gonvers et al. [24] found higher incidence of cataract development in older patients (14% of the younger patients [10 to 40 years] versus 37% of the older patients [41 to 50 years]). Choi et al. [25], in a 10-year clinical study (*n* = 110), found lens opacities in 21 eyes (12.1%) and the mean age at which lens opacity was first observed was 48.7 ± 7.1 years. They found a difference in the preoperative age between the clear lens group and lens opacity group as well as a higher cumulative incidence of lens opacity in patients with higher preoperative age. In addition, their mean vault value was significantly lower in older patients 10 years after ICL implantation, whereas it did not differ significantly at 6 months. In the same way, Lindland et al. [26] also reported lower vault in older patients over a follow-up of about 5 years. However, all these studies evaluated patients implanted with older ICL models. With the present V4c ICL, recent long-term studies [13, 14] and meta-analysis reported no postoperative complications [1, 2]. Kamiya et al. [3] and Takahashi et al. [4], in an older patient sample (with 6 months of follow-up) similar to ours, did not report either asymptomatic or symptomatic cataract formation. Thus, it seems that a new lens design (which improves the circulation of the aqueous humor to the anterior surface of the crystalline lens [13]) and new anterior-segment imaging systems being used (providing accurate measurements of several parameters mandatory for ICL size calculation) are essential factors that may contribute to the zero-incidence of cataract formation. With accurate vault measurement and controlled follow-up, the risk for lens opacity in patients who have ICL implantation over 40 years seems to be minimal. However, we need to bear in mind that our study's sample is small and that the follow-up was short. A large cohort of patients with longer follow-up periods is necessary to confirm our outcomes. Some limitations of our study are, in addition to the small sample and short follow-up, the lack of measurement of near vision and crystalline lens rise, which should be included in future studies.

In conclusion, the V4c ICL implanted in patients over 40 years showed good visual outcomes, resulting in improved UDVA and CDVA after surgery. The refractive outcomes were also successful, confirming the procedure's high predictability. Our results are in good agreement with previous studies performed in younger samples. So, this lens may be considered a good procedure to correct myopia and myopic astigmatism in older patients. Future studies with larger samples and longer follow-up periods are needed to fully characterize this lens' outcomes in this specific patients' age range.

## Figures and Tables

**Table 1:** Preoperative patient demographics and ICL characteristics.

	Mean ± SD	Range [min, max]
Age (years)	43.52 ± 4.49	[40, 56]
Refraction sphere (D)	−8.18 ± 2.66	[−13.5, −2]
Refraction cylinder (D)	−1.55 ± 1.18	[−4.0, 0]
Spherical equivalent (D)	−8.46 ± 3.31	[−14.88, −1.88]
Corneal thickness (*μ*m)	535.18 ± 36.66	[452, 580]
CDVA (decimal)	0.88 ± 0.18	[0.40, 1.00]
ACD (mm)	3.07 ± 0.26	[2.67, 3.59]
WTW (mm)	12.16 ± 0.41	[11.30, 12.70]
ATA (mm)	12.07 ± 0.39	[11.50, 12.79]
ECD (cells/mm2)	2568 ± 332	[2004, 3297]
IOP (mmHg)	16.23 ± 3.61	[10, 24]
ICL sphere (D)	−9.52 ± 2.65	[−15.0, −1.0]
ICL cylinder (D)	2.65 ± 1.23	[1, 4.5]
ICL size (mm)	12.96 ± 0.35	[12.10, 13.20]

ICL: implantable Collamer lens; SD: standard deviation; D: diopters; CDVA: corrected-distance visual acuity; ACD: anterior chamber depth; WTW: white-to-white; ATA: angle-to-angle; ECD: endothelial cell density; IOP: intraocular pressure.

**Table 2:** Studies reporting data for the V4c ICL with 1 year of follow-up: year of publication, sample age, and preoperative spherical equivalent (SE) (mean, standard deviation, and range).

Author	Year	Eyes (patients)	Age (ys)	SE (D)
Lisa et al. [5]	2015	147 (80)	30.4 ± 4.8 (20 to 40)	−8.80 ± 2.60 (−2.75 to −17.50 sph 0 to −3.00 cyl)
Karandikar et al. [6]	2015	34 (34)	26.13 ± 3.8 (NR)	−9.24 ± 2.4 (NR)
Ganesh et al. [7]	2017	NR (NR)	26.4 ± 2.4 (NR)	−5.98 ± 1.15 (NR)
Pjano et al. [8]	2017	28 (16)	28.21 ± 4.06 (21 to 35)	−9.52 ± 3.69 (NR)
Kamiya et al. [9]	2018			
Low-to-moderate myopia		57 (57)	34.8 ± 7.4 (20 to 57)	−4.29 ± 1.31 (−0.50 to −5.88)
High myopia		294 (294)	33.6 ± 7.3 (18 to 54)	−10.13 ± 2.64 (−6.00 to −18.63)
García-de la Rosa et al. [10]	2018	76 (42)	27.4 ± 5.14 (20 to 39)	−11.94 ± 3.51 (−7.50 to −22.88)
Niu et al. [11]	2019	40 (31)	32.45 ± 6.85 (20 to 42)	−14.03 ± 4.46 (−7.50 to −25.75)
Sachdev et al. [12]	2020	203 (NR)	24 ± NR (22 to 28)	−9.44 ± 4.20 (−1.75 to −21)
Current study	2020	33 (21)	43.52 ± 4.49 (40 to 56)	−8.46 ± 3.31 (−1.88 to −14.88)

**Table 3:** Visual and refractive outcomes, intraocular pressure (IOP), endothelial cell density (ECD), and vault of the different studies considered (mean, standard deviation, and range) NR: not reported.

Author	UCVA (logMAR)	CDVA (logMAR)	Safety index	Efficacy index	±0.50D (% eyes)	±1.00D (% eyes)	SE (D)	IOP (mmHg) (eyes >21)	ECD (cells/m^2^) (% loss)	Vault (*μ*m)
Lisa et al. [5]	0.028 ± 0.055	0.003 ± 0.013	1.04	1.00	93.9	100	−0.14 ± 0.26	12.4 ± 1.4 (0 > 20)	2650 ± 438 (1.7)	405.5 ± 184.7 (100 to 980)
Karandikar et al. [6]	NR	NR	1.15	1.6	57.12	98.12	−0.19 ± 1.18	19.1 ± 1.3	NR (7.1)	628.2 ± 300.1 (NR)
Ganesh et al. [7]	−0.022 ± 0.021	−0.071 ± 0.079	1.24	1.12	90	100	−0.164 ± 0.20	NR	2808 ± 315 (9.0)	NR
Pjano et al. [8]	0.76 ± 0.16 (decimal)	0.79 ± 0.14 (decimal)	1.25	1.2	NR	NR	−0.21 ± 0.27	14.96 ± 1.7	2512 ± 127 (5.5)	NR
Kamiya et al. [9]										
Low-to-moderate myopia	−0.17 ± 0.14	−0.21 ± 0.10	NR	NR	93	98	−0.12	13.1 (0)	NR (0.1)	NR
High myopia	−0.16 ± 0.09	−0.21 ± 0.08	NR	NR	94	99	0.02	13.6 (0)	NR (0.1)	NR
García-de la Rosa et al. [10]	0.12 ± 0.12	0.05 ± 0.08	NR	NR	85	86	−0.06 ± 0.77	NR	NR	449 ± 180 (NR)
Niu et al. [11]	0.89 ± 0.30 (decimal)	1.00 ± 0.27 (decimal)	1.33	1.14	69	92	−0.67 ± 1.29	15.15 ± 2.57 (0)	2963 ± 396 (8.38)	380 ± 152 (90 to 700)
Sachdev et al. [12]	0 ± NR	0 ± NR	NR	NR	94.09	96.09	−0.12 ± NR	NR	NR	NR
Current study	0.88 ± 0.16 (decimal)	0.96 ± 0.09 (decimal)	1.09	1.00	90.6	93.9	−0.09 ± 0.47	15.27 ± 3.03 (0)	2516 ± 234 (2.04)	320 ± 136 (96 to 603)

UCVA: uncorrected distance visual acuity, CDVA: corrected distance visual acuity, SE: spherical equivalent, and NR: not reported.

## Data Availability

All data generated or analysed during this study are available within the article.
